# Editorial: Systems biology approaches to psychiatric and psychological disorders: unraveling the complexities

**DOI:** 10.3389/fgene.2025.1547943

**Published:** 2025-01-28

**Authors:** Ruoting Yang, Bernie J. Daigle, Ryan Rampersaud, Katharina Schultebraucks

**Affiliations:** ^1^ Medical Readiness Systems Biology, Walter Reed Army Institute of Research, Silver Spring, MD, United States; ^2^ Departments of Biological Sciences and Computer Science, University of Memphis, Memphis, TN, United States; ^3^ Department of Psychiatry and Behavioral Sciences, University of California San Francisco School of Medicine, San Francisco, CA, United States; ^4^ Department of Psychiatry, New York University Grossman School of Medicine, New York, NY, United States

**Keywords:** systems biology, psychological disorders, computational biology, molecular assay, biomarker

Trauma, whether psychological, physiological, or a combination of both, remains a profound driver of molecular, cellular, and behavioral changes across organisms. The articles in this Research Topic delve into the intricate interplay between genomic, genetic, epigenetic, metabolic, proteomic, and environmental factors contributing to trauma responses and long-term pathophysiological outcomes. This editorial synthesizes key findings from studies exploring novel diagnostic tools, molecular signaling mechanisms, and therapeutic strategies, with a focus on their potential translational applications.

One central theme in this Research Topic is the role of metabolites as intermediaries of trauma response. Gary et al. highlighted dysregulated pathways in acute stress disorder (ASD), including amino acid metabolism and lipid signaling, revealing potential metabolic biomarkers for early diagnosis and intervention. Similarly, Patel et al. examined how dietary polyunsaturated fatty acids (PUFAs) modulate neuronal resilience to traumatic brain injury (TBI) and stress, implicating diet as a critical factor in mitigating visual and neuronal deficits. Transcriptomic analyses showed that DHA-enriched diets activate pathways such as SNARE signaling, endocannabinoid synapse pathways, and synaptic long-term depression, while suppressing inflammatory cytokine signaling (e.g., IL-6, IL-8) and ferroptosis, underscoring the interaction between PUFA treatment, TBI, and brain signaling networks. These findings emphasize the need to consider both endogenous and environmental influences in understanding stress resilience and vulnerability.

Another key focus is the gut-brain axis and its role in stress and mood disorders. Chakraborty et al. reviewed how microbial metabolites mediate the host’s response to environmental stressors, suggesting a bidirectional relationship influencing neuroinflammation and behavior. Furthering our understanding of the gut-brain axis in the context of mental health, Pinakhina et al. investigated the intronic variant rs521851 in the MAGI2 (S-SCAM) gene significantly associated with depression symptoms in individuals with a high risk of eating disorders, highlighting its influence on gut-brain axis dysregulation. Their results underscore the genetic underpinnings of psychiatric conditions and reinforce the interplay between genetic predisposition and environmental stressors. Such insights underscore the potential of targeting microbiome-related pathways and considering genetic factors in addressing trauma-related disorders.

Beyond the gut-brain axis, researchers also employed cutting-edge tools to investigate the complexities of stress response systems. Parker et al. employed neural ordinary differential equations (NODEs) to model the hypothalamic-pituitary-adrenal (HPA) axis dynamics, offering a machine-learning-based framework to predict stress responses in depressive disorders. This innovative approach highlights the growing importance of integrating computational models with biological data to enhance diagnostic accuracy and therapeutic precision.

In summary, this Research Topic underscores the complexity and interconnectedness of molecular systems in trauma and stress responses. By fostering interdisciplinary collaboration and innovative methodologies, these studies pave the way for more holistic approaches to understanding and mitigating the impacts of trauma on health.

